# Simulation Improves Emergency Medicine Residents’ Clinical Performance of Aorta Point-of-Care Ultrasound

**DOI:** 10.5811/westjem.18449

**Published:** 2024-02-09

**Authors:** Brandon M. Wubben, Cory Wittrock

**Affiliations:** University of Iowa Hospitals and Clinics, Department of Emergency Medicine, Iowa City, Iowa

**Keywords:** point-of-care, ultrasonography, simulation, abdominal aortic aneurysm, POCUS, emergency medicine

## Abstract

**Purpose:**

Using point-of-care ultrasound (POCUS) to diagnose abdominal aortic aneurysm (AAA) is an essential skill in emergency medicine (EM). While simulation-based POCUS education is commonly used, the translation to performance in the emergency department (ED) is unknown. We investigated whether adding case-based simulation to an EM residency curriculum was associated with changes in the quantity and quality of aorta POCUS performed by residents in the ED.

**Methods:**

A case-based simulation was introduced to resident didactics at our academic, Level I trauma center. A case of undifferentiated abdominal pain was presented, which required examination of an ultrasound phantom to diagnose an AAA, with a hands-on didactic. We compared the quantity, quality, and descriptive analyses of aorta POCUS performed in the ED during the four months before and after the simulation.

**Results:**

For participating residents (17/32), there was an 86% increase in total studies and an 80% increase in clinical studies. On an opportunity-adjusted, per-resident basis, there was no significant difference in median total scans per 100 shifts (4.4 [interquartile range (IQR) 0–15.8 vs 8.3 [IQR] 3.3–23.6, *P* = 0.21) or average total quality scores (3.2 ± 0.6 vs 3.2 ± 0.5, *P* = 0.92). The total number of limited or inadequate studies decreased (43% vs 19%, *P* = 0.02), and the proportion of scans submitted by interns increased (7% vs 54%, *P* = < .001).

**Conclusion:**

After simulation training, aorta POCUS was performed more frequently, and ED interns contributed a higher proportion of scans. While there was no improvement in quantity or quality scores on a per-resident basis, there were significantly fewer incomplete or limited scans.

Population Health Research Capsule What do we already know about this issue? *Simulation has increasingly been used to prepare EM residents for less common conditions, such as diagnosing abdominal aortic aneurysm using POCUS.*

What was the research question?

*Does case-based POCUS simulation affect the quantity or quality of aorta POCUS that residents perform in the ED?*

What was the major finding of the study?

*Aorta POCUS increased 86%, and the number of limited or inadequate studies decreased (43% vs 19%, P = 0.02).*

How does this improve population health?

*Aorta POCUS simulation training may help physicians who less frequently encounter aortic aneurysm to identify this time-sensitive condition.*

## INTRODUCTION

Point-of-care ultrasound (POCUS) of the aorta to diagnose abdominal aortic aneurysm (AAA) is a core emergency ultrasound application and an essential component of emergency medicine (EM) residency education.^1^ Emergency department (ED) POCUS has previously been shown to have excellent performance characteristics for the evaluation of AAA.^2^ Simulation is a commonly used educational tool for resident ultrasound education, but a scoping review found the majority of prior studies examined changes in confidence, knowledge, and skills rather than objective clinical performance.^3^ However, simulation in addition to clinical training has been shown to be effective at translating to clinical performance in other specialties, such as for obstetrics and gynecology residents learning transvaginal ultrasound.^4^

It is critical that emergency physicians learn to quickly identify AAA at the bedside, as this is a time-sensitive and potentially deadly diagnosis requiring a goal time from presentation to emergency surgery of <90 minutes.^5^ We aimed to investigate whether the addition of case-based ultrasound simulation to the existing EM residency curriculum was associated with an increase in the quantity and quality of aorta POCUS performed on ED patients.

## MATERIALS AND METHODS

### Design, Setting, and Intervention

At our academic, Level I trauma center with a three-year EM residency program and advanced practice provider training program, EM residents have existing simulation-based learning built into their monthly academic conference. However, the use of POCUS is not typically incorporated into simulation at our institution. In Spring 2023, we introduced a new simulated case requiring the use of POCUS for diagnosis of AAA in the setting of undifferentiated abdominal pain. During simulation-based learning, residents are divided into groups of 5–8 residents of varied postgraduate year (PGY) and cycle between the simulation session and other educational activities. In addition to verbal prompts regarding case history, physical exam findings, and patient responses to interventions, residents were asked to use a cart-based ultrasound system (Sonosite PX, Fujifilm, Bothell, WA) to examine an ultrasound phantom abdomen (41903–000, Kyoto Kagaku, Japan) with multiple findings including an infrarenal AAA with intermural thrombus, free abdominal fluid, and normal bowels and renal system. The case concluded with a hands-on didactic led by a POCUS fellowship-trained emergency physician and included time at the end of the simulation for hands-on scanning by residents.

### Ultrasound Study Review and Outcomes

All ultrasound studies performed in the ED are submitted through a quality assurance workflow for review by a team of POCUS fellowship-trained faculty. All residents who participate receive credit for performing the POCUS. The submitting resident who performed the POCUS completes a worksheet describing the findings, interpretation, and study limitations. Studies are marked complete if residents indicate that a view was obtained of the suprarenal aorta, infrarenal aorta, and iliac bifurcation and incomplete if one or more of these views was not obtained. Images and worksheets are then sent for review and signature to the faculty caring for the patient with the resident. Faculty can either place the study in an educational archive (if they did not use the study for medical decision-making) or a clinical archive (if they used the study for medical decision-making and wish for the images to transfer to the patient’s health record).

Studies are reviewed for quality and assigned a quality score from 1 (worst) to 5 (best) as well as notation of any false positives or false negatives, with EM ultrasound faculty serving as the gold standard. Quality scores 1 and 2 are considered insufficient for diagnosis, with scores of 3–5 considered adequate. We examined cumulative measures of sensitivity and specificity before and after the simulation.

### Analysis

We compared the median number of aorta POCUS studies that EM residents performed in the ED in the four months prior to the simulation session to the median number of exams performed in the four months following the simulation session. These quantities were reported as scans performed per 100 shifts per resident and compared using Wilcoxon signed-rank test. We compared mean quality scores over the four months prior to the intervention to the quality scores over the four months after the intervention as described above with the quantity of exams using a paired *t*-test. Proportions of limited studies and training year distribution were compared with Pearson chi-square. The significance level of all tests was set to 0.05 with Bonferroni correction applied where appropriate. Analysis was performed in SPSS for Macintosh, v 28.0 (SPSS Inc, Chicago, IL). This study received institutional review board approval for waiver of signed informed consent.

## RESULTS

Over half of residents 17/32 (53%) participated in the simulation session and had at least one clinical shift before and after the simulation session. The distribution of the participating residents as primary study authors is demonstrated in the Table, with a significant increase in the proportion of aorta POCUS submitted by interns. Overall, there was an 86% increase in total studies and an 80% increase in clinical studies after the session. However, when comparing on a per-resident basis while adjusting for clinical opportunities, there was no significant difference in median total scanning frequency per 100 shifts (4.4 [interquartile range (IQR) = 0–15.8] vs 8.3 [IQR = 3.3–23.6], *P* = 0.21). There was also no significant change in average total quality scores on a per resident basis (3.2 ± 0.6 vs 3.2 ± 0.5, *P* = 0.92).

**Table.** Number of aorta point-of-care ultrasound studies submitted before and after simulation training, stratified by training level of the primary study author by archive. (*): P = < .05 with Bonferroni correction.

	Clinical			Educational			Total		
	Pre	Post	*P* (*X* ^2^)	Pre	Post	*P* (*X* ^2^)	Pre	Post	*P* (*X* ^2^)
Level			.34			<.001			<.001
APP	1 (20%)	1 (11%)		0 (0%)	1 (2%)		1 (4%)	2 (4%)	
PGY-1	0 (0%)	3 (33%)		2 (9%)	25 (58%)	*	2 (7%)	28 (54%)	*
PGY-2	3 (60%)	2 (22%)		12 (52%)	5 (12%)	*	15 (54%)	7 (14%)	*
PGY-3	1 (20%)	3 (33%)		9 (39%)	12 (28%)		10 (36%)	15 (29%)	

*APP*, advanced practice provider resident; *PGY*, postgraduate year.

There were no false negative or false positives using faculty review of images as the gold standard. There were no differences in the proportion of studies with agreement vs disagreement with the resident interpretation (100% vs 96%, *P* = 0.29). There was a decrease in the total number of limited or inadequate studies (12/28 (43%) vs 10/52 (19%), *P* = .02 [*X* ^2^]). There was no significant change in the proportion of clinical studies submitted as “limited” or “inadequate” (2/5 (40%) vs 4/9 (44%), *P* = 0.87 [*X* ^2^]), but the number of educational studies submitted as “limited” or “inadequate” improved (10/23 (44%) vs 6/43 (14%), *P* = <.001).

## DISCUSSION

Overall, the total number of aorta POCUS studies performed in the ED after the simulation increased, albeit without a demonstrable change in quantity or quality rating on a per-resident basis. However, there were a number of positive findings, which support the inclusion of ultrasound simulation in residency training, including a significant increase in the proportion of studies contributed by interns and a significant decrease in the proportion of studies that were incomplete or limited.

It seems unlikely that the significant increase in intern POCUS studies was due to content mastery based on compounding clinical experience alone. Aorta POCUS is one of the applications requiring the most experience to gain proficiency, and with previously demonstrated plateau points in interpretation and acquisition at 66 studies and 84 studies, respectively, which were not approached by anyone in our study.^6^ The same study found that aorta POCUS quality actually decreases initially with increasing number of scans before it eventually improves above baseline, which may be contributing to the absence of improvement in median quality scores seen in our study.^6^

Much of the published research regarding POCUS simulation reports outcomes related to the assessment of learner experience and skill performance outside the clinical context.^7^ ^,^ ^8^ While these outcomes are important, there is a desire to assess more translational outcomes resulting from simulation interventions.^9^ ^,^ ^10^ There are few translational studies available for direct comparison to the current study. Our simulation experience was delivered as a single session, which is less time-intensive than a prior study of EM interns that found positive clinical effects of simulation-based mastery learning on performance of focused assessment with sonography in trauma.^11^ Outside of EM, a randomized trial of ultrasound simulation for obstetrics and gynecology residents that was also more longitudinal than the current study also found positive clinical effects of early simulation training, in addition to clinical practice in first-year residents.^4^ Further study is needed to determine whether the case-based simulation approach in our study would be more successful if the training were more time intensive, more longitudinal, and most targeted toward junior learners.

## LIMITATIONS

Our findings are subject to the limitations of a before-and-after study, including the possibility that other factors may have contributed to the observed changes other than the simulation; however, we are not aware of any other targeted effort to educate our residents about aorta POCUS during the study period, and believe it is likely most changes were associated with the simulation. Second, this was a study of residents whose categorization of study intent was subject to their signing faculties’ preferences. Therefore, we included both educational and clinical archive studies to provide a fair portfolio of each resident’s work, although some educational studies may have not been intended for patient care. Third, EM residents do not get to choose which patients they take care of in the ED (because emergencies are unplanned and unpredictable); so some residents likely had slightly more exposure to patients with indications for aorta POCUS than others. In addition, while we were able to adjust for clinical opportunities based on time spent in the ED, residents often see more patients per hour as they advance through training, and we were unable to account for total patients seen during the study period. Even considering these limitations, we believe the data presented provides an accurate real-world assessment of scanning frequency and quality on ED patients by ED residents.

## CONCLUSION

In the four months following a case-based simulation to diagnose abdominal aortic aneurysm using point-of-care ultrasound, the proportion of aorta POCUS studies performed in the ED by interns increased significantly, and the proportion of studies that were incomplete or limited significantly decreased. While there was no overall increase in the median number of scans or mean quality scores when adjusted for clinical opportunities on a per-resident basis, among residents as a whole there was an 86% increase in submitted aorta POCUS studies.
